# Multi-agent systems in epidemiology: a first step for computational biology in the study of vector-borne disease transmission

**DOI:** 10.1186/1471-2105-9-435

**Published:** 2008-10-15

**Authors:** Benjamin Roche, Jean-François Guégan, François Bousquet

**Affiliations:** 1 UMR 2724 Génétique et Évolution des Maladies Infectieuses, IRD-CNRS-Université de Montpellier I, Montpellier, France; 2 French Center on Globalization and Infectious Diseases, French School of Public Health, Montpellier, France; 3 Centre International de Recherche en Agronomie pour le Développement, Montpellier, France

## Abstract

**Background:**

Computational biology is often associated with genetic or genomic studies only. However, thanks to the increase of computational resources, computational models are appreciated as useful tools in many other scientific fields. Such modeling systems are particularly relevant for the study of complex systems, like the epidemiology of emerging infectious diseases. So far, mathematical models remain the main tool for the epidemiological and ecological analysis of infectious diseases, with SIR models could be seen as an implicit standard in epidemiology. Unfortunately, these models are based on differential equations and, therefore, can become very rapidly unmanageable due to the too many parameters which need to be taken into consideration. For instance, in the case of zoonotic and vector-borne diseases in wildlife many different potential host species could be involved in the life-cycle of disease transmission, and SIR models might not be the most suitable tool to truly capture the overall disease circulation within that environment. This limitation underlines the necessity to develop a standard spatial model that can cope with the transmission of disease in realistic ecosystems.

**Results:**

Computational biology may prove to be flexible enough to take into account the natural complexity observed in both natural and man-made ecosystems. In this paper, we propose a new computational model to study the transmission of infectious diseases in a spatially explicit context. We developed a multi-agent system model for vector-borne disease transmission in a realistic spatial environment.

**Conclusion:**

Here we describe in detail the general behavior of this model that we hope will become a standard reference for the study of vector-borne disease transmission in wildlife. To conclude, we show how this simple model could be easily adapted and modified to be used as a common framework for further research developments in this field.

## Background

In a reductive way, computational biology is often assimilated to the study of genomes, genetic networks, or other subjects at intra-host level. In this field, a deep insight has been reached in phylogenetic reconstructions for instance [1]. However, oddly enough, computational biology is rarely associated with the study of larger systems such as ecosystem dynamics. More specifically, despite a huge amount of mathematical models which increasing complexity, little work has been done in computational biology to understand the transmission of disease agents in natural populations and communities of hosts. In the current context of disease emergence [2], computational modeling could become the tool of choice for dissecting the complexity of mechanisms that can be at work to produce disease outbreaks.

So far, mathematical models have been mainly used for epidemiological modeling. The prolific development of mathematical models has been mostly caused by the existence of a standard model, the so-called SIR (or SEIR) model. This "toy" model has been extensively studied and applied [3,4] and it has proved to be particularly accurate for many different biological questions, both in epidemiology [5,6] and evolution [7,8]. The strength of this model relies on its adaptability [3] and simplicity of formulation, despite its current complexity in generated patterns [9].

Spatial modeling suffers of a dramatic lack of this type of reference models except for directly-transmitted diseases. These diseases, where pathogens are transmitted between individuals by close contacts, can be spatially analyzed using an implicit standard model based on cellular automata [10-13]. Some more complex models exist for avian influenza [14], but they have been done in a prospective way. Other kinds of diseases have been poorly studied in a spatial context, especially vector-borne diseases. These pathogens are transmitted between reservoir individuals, which are generally vertebrate hosts, by the bite of one vector individual, which is generally an haematophagous insect, such as a tick or a mosquito. Despite an huge literature in a non-spatial context [15,3,16], spatial models are rare for vector-borne disease [17-19]. However, these models, built upon the framework of cellular automata, involve very simple systems with only one vector and one reservoir species, when typically vector-borne diseases can locally involve several vector and reservoir species [20]. Hence, current approaches, *i.e*. mathematical modeling or cellular automata modeling, seems to be unable to study vector-borne diseases with a large host spectra within an heterogeneous environment. We thus develop a spatial model, based upon the framework of multi-agent systems [21], for multi-host vector-borne diseases in realistic environments.

Four main categories of spatial models can be distinguished [11,12], depending on the kind of tools used and the biological questions considered. The first one is the reaction-diffusion system that is based on physical processes, and has been mainly used for vector-borne diseases and more specifically for vector spreading [22]. It assumes a continuous diffusion of vectors but it is likely to be not the most suitable for realistic environments. The second is a model based on the network theory [23]. This theory has supported studies on the impact of transport networks in disease dynamics [24,25] and focuses on the properties of the network studied. The third method is based on metapopulation theory [26]. This conceptual framework has been widely used for the study of spatio-temporal dynamics of infectious diseases, especially childhood diseases [27,28]. However, despite that this theory has been widely applied in conceptual frameworks, it focuses mainly on a nation-wide scale [29,30] or at least on large-surface areas. The last available tools are multi-agent systems [31], that are based on cellular automata [32], but that are applied in a more "cognitive" way. Boundaries between multi-agent and cellular automata often seem mostly philosophical and semantic. An extensive literature on multi-agent systems exists, especially in social sciences [33] and ecology [21]. In epidemiology, despite some individual-based models for directly-transmitted diseases [34,14], multi-agent systems have not yet been used to study vector-borne disease dynamics in spatial contexts.

Here, we describe a simple and robust multi-agent model which can be easily extended to the study of more complex situations. Then, we show three extensions of our model in the section "Examples of adaptability". The first one is the possibility to integrate a real landscape derived from a Geographical Information System (GIS) software. Since this kind of input often requires highly-intensive computing, we have then developed a parallel version of our model. Finally, we show how the evolutionary dynamics dimension of host-parasite systems can be integrated into our model.

## Methods

### Philosophy of this model

Earlier individual-based systems [14,34] were quite complex and used the computational framework to produce very complicated models. The main target of these models was to make predictions about possible future dynamics of a given disease. We followed a totally different approach in order to build a very simple model, and in this paper we will: (i) give a clear and detailed description of our simple spatial model to be used as a "reference" for vector-borne diseases, (ii) define the core mechanisms of vector-borne diseases in a spatial context that, hopefully, will be used as reference for future studies (Additional file 1, section 2.1) and (iii) explain how this model could be easily adapted to other disease situations. The theoretical analysis putted in the supplementary informations has been done with an high (and unrealistic) value of pathogen transmission to allow us an analysis without an huge number of host individuals, both vectors and reservoirs.

Our epidemiological framework was inspired by the classical model proposed first by Kermarck and McK-endrick [35] and most popularized by Anderson and May [3]. Accordingly, we classified the two types of hosts, i.e. vectors and reservoirs, involved in disease transmission into four categories. Individuals are born in a "Susceptible", not infected, state. Upon infection, individuals become "Infected", but at this stage they are unable to infect other hosts. Then, after a given "latency period", those individuals become "Infectious" and can infect other subjects. Finally, after an "infectious period", individuals become "Recovered" and they are immune against the disease agent, with the exception of vector individuals which remain infectious until their death [36]. However, some vector-borne pathogen, like *Plasmodium falciparum*, could develop an immunity in mosquito species. To remove this limitation, an adaptation of "step" function is needed. We assume that an immunity is developed into reservoir individuals despite that some pathogens do not lead to immunity for reservoir individuals [3]. To remove this immunity, the field "infectious period" could be filled with zero. It is important to note that this model has been developed to study, in its current form, a virus transmitted by mosquitoes species. However, our model is easily adaptable for other kind of vector-borne diseases as we discuss for different assumptions.

This model could be analyzed in both ways : (i) a conceptual way to study, for instance, the structure of spatio-temporal dynamics of vector-borne diseases and (ii) an applied way by integrating real data, from a GIS for instance, which allow us to track, and eventually to predict, the spatio-temporal dynamics of a given disease in a given environment, like West Nile Fever in Southern France for instance.

From an ecological point of view, the present model is supported by a strong theoretical and empirical framework [37]. We assumed that host species were distributed spatially and clustered within different specific ecological habitats. Hence, host species within a habitat could not leave their original habitat except within a given range of tolerance value. This important parameter will be further developed, especially in Additional file 1, section 2.2.

We developed this model in order to be able to study dynamics of vector-borne diseases when multiple host species are involved (Additional file 1, section 2.3); however it might also be applied to assess other scientific questions with similar objectives.

### Components of the multi-agent system

We used an oriented-object approach in which a "class" is an abstract pattern of a physical representation and an "object" an instance of a class. Each class could be linked to other classes by "attributes", which represent the properties of a class, or by "methods", which represent the different functions applicable to each object. Our model was implemented with the support of the SWARM platform [38] and was developed in Java language.

**Figure 1:**
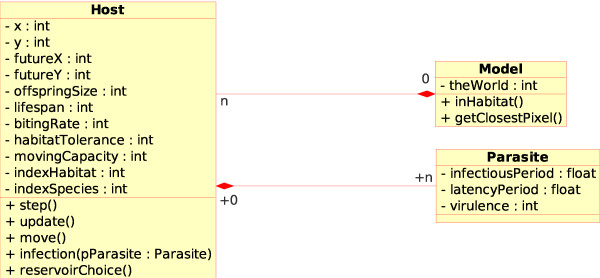
**Static UML modeling of our model**. The "Model Swarm" class, which contains the representation of the virtual world where the pathogen agent circulates, contains links to all "Host" objects. The "Host" object can contain, or not, one "Parasite" object if Host is infected, infectious or has been infected. All attributes and functions are detailed in the main text.

The structure of our model, i.e. the relationships among classes, is described in Figure 1. This UML (Unified Modeling Language) modeling shows the different parts of our model. A "Host", which could be a vector or a reservoir individual, can host one "Parasite" agent. All "Host" objects are contained within a "World" object, which comprises the computing representation of that physical world. "Parasite" agents are also contained within this "World" object. All these parts, and the most important functions and attributes used, will be detailed hereafter.

#### Parasite

The "Parasite" class includes different characteristics related only to the disease agent and the illness, such as "Infectious Period", "Latency Period" and "Virulence". These features can be applied to both vector and reservoir species individuals. Since disease characteristics are in reality a by-product of host and parasite life history-traits, this simplification leads to some limitations of the model, as we assume that parasite characteristics are exactly identical within vectors and reservoirs species. This approximation is acceptable for diseases with multiple vector and reservoir species if parasite's influences are similar among the different host species [3,39]. However, this approximation needs to be reduced if we want to address disease transmission by different strains of a given parasite. Since the main goal of the current work is to deal with a simple disease system without evolutionary dynamics, this issue will be discussed in the final "Examples of adaptability" section.

#### Host

The "Host" class is the most important class in our model and has different groups of attributes. The first one concerns the geographic localization of individuals. Hence, host contains "x and "y" attributes that represent its geographical localization. For update purposes, we added also the "future X" and "future Y" attributes, which contain future positions of a given host individual. These attributes will be dynamically updated over time. The second group concerns species characteristics that do not change over time. Within this group, we can distinguish ecological life-history traits, such as "Offspring Size", "Lifespan" and "Biting Rate", and spatial features, such as "Moving Capacity" and "Habitat Tolerance". "Moving Capacity" represents the area in pixels in which one host is allowed to move, and "Habitat Tolerance", a core parameter of our model, quantifies for each host species how far, in pixels, one host is allowed to diffuse outside its original habitat. To distinguish between vector and reservoir individuals, the "Biting Rate" attribute will be equal to "-1" for reservoir individuals and to the corresponding biting rate for vectors. This attribute is assumed to be constant for mosquito-borne pathogens, but it could evolve dynamically for tick-borne disease regarding the abundance of reservoir hosts involved. To study this kind of vector-borne pathogen, this attribute have to be replaced by a function involving the reservoir abundance.

We could have distinguished two different "Vector" and "Reservoir" classes which inherit from the "Host" class. However, we think that new classes inheriting from a super-class should be created only in the presence of certain conditions. The first case is when the number of inheriting classes is high or could be high. This is clearly not our case since only two classes are definitively needed. The second case is when the number of functions, which have to be inherited from a super-class, is high. In our case, only the function "recovering" would have to be developed differently and the function "Move" partly in a different way. Moreover, many functions of the "Model Swarm" class use general properties of the host and do not need to know whether a given individual is a vector or a reservoir. Therefore, we chose to integrate "Vector" and "Reservoir" particularities into a single "Host" class.

Finally, the "Index Habitat" and "Index Species" attributes are used for computational purposes. These different characteristics will be the same among all individuals within a given host species. However, a given "Index Species" could have links with different "Index Habitats" (see Additional file 1, section 3, to understand how files are loaded). As already said for the "Parasite" class, host reactions (e.g. immune responses, mechanical responses,...) against the parasite are not considered here, and all host-parasite interactions are accepted as being static in the "Parasite" class but not for the "Competence" parameter. This parameter is integrated into the "Host" class and represents the probability for a host individual to become "Infectious" after a contact with an "Infectious" individual.

#### Landscape of habitats

Finally, landscape will be simply an array with the size of the virtual world. This array will be contained into a class, "Model Swarm", which contains different functions that impact on the landscape. This is an array of Integer values that correspond to indexes of habitats that will be linked to the Host's "Index Habitat" attribute. Thus, with these two parameters, we can characterize the whole spatial distribution of the host species, and will be able to address any landscape configuration. This array could be assimilated to a picture that describes the category of habitats occurring at each pixel.

### Dynamical interactions between components

So far we have defined the static part of our model. Now we need to address the dynamic interactions among all these attributes. Indeed, each object will interact with all the others both in space and time using simple dynamical functions. To keep our model as simple as possible, we have modeled only three functions that are scheduled by a main algorithm and are implemented in the "Host" class.

#### Host with landscape: Moving function

There are two options in the moving function. The first one is a basic type of movement and each individual has a given "Moving Capacity" that will allow that subject to move from its initial position to ((*MovingCapacity *+ 1) * 2)^2 ^new locations. At each time step, a new position will be randomly selected. If it is not within the individual's habitat (with respect to the "Tolerance value"), the nearest position into the habitat will be then selected. We begin with movement with a square, but this function could be easily modified to take into account different behaviors.

The other option concerns only the vectors. Each vector individual has a "Biting Rate" which is converted into the probability of biting at each time step and is equal to *1*/*BitingRate *when "Biting Rate" is expressed with the time step used in simulations. When this event is selected, the number of available reservoir individuals in ((*MovingCapacity *+ 1) * 2)^2 ^possible positions is computed. A reservoir individual is then chosen randomly from a uniform distribution, and the vector will move to this selected position and bite the chosen reservoir individual for a potential transmission between vector and reservoir.

#### Host with parasite: Infection function

Potential infection is the second function, and it could be involved at each time step. Each individual, both from vector and reservoir species, has a "Competence" value, which represents the probability that an individual becomes infectious after an infectious contact. Hence, a random number will be generated according to a uniform law and, if this number is inferior to the "Competence Value", the "next Parasite" field receives a "parasite" input. At each time step, "current Parasite" has a *1*/*latencyPeriod *probability to receive the "next Parasite" object if this object is not null. If successful, "next Parasite" will receive a "null" value. Similarly, at each time step, "past Parasite" has a *1*/*infectiousPeriod *probability to receive a "current Parasite" object. Hence, the "next Parasite", "current Parasite" and "past Parasite" fields will determine the infectious status as follows: (i) if the three fields are "null", that individual is in the "Susceptible" status; (ii) if "next Parasite" is not null and the others are null, that subject is "Infected", but not infectious (iii) if "current Parasite" is not null and the others are null, the individual is "Infectious" and can transmit the disease agent, and finally (iv) when "past Parasite" is not null and the others are null, the individual is "Recovered" and can not be infected again.

**Table 1:** Summary of infection status

Field not null	Status
No field	Susceptible
next Parasite	Exposed
current Parasite	Infectious
past Parasite	Recovered

#### Host: Birth and death functions

The last important functions concern demography. Each individual from a same species has an "Offspring Size" mean which represents the number of new hosts created at each birth event. To simplify, we assume that each "Host" object has, on average, only one birth event during its life. Hence, at each time step, there is a *1*/*lifeSpan *probability that new "Offspring Size" "Host" objects will be created for each individual. In the same way, individuals die with a probability equals to *1*/*lifeSpan *at each time step. To keep our populations constant, as we assumed in the rest of this paper, we set "Offspring Size" to one. Thus, on average, each host will produce one new host before its death. This assumption could be easily relaxed by adding a specific attribute representing a birth rate, which could be also a function to cope with dynamical demography.

#### Main algorithm

Finally, each individual has a main algorithm which schedules all the previously described different functions. This algorithm is split in two main functions: "Step" and "Update". The "Step" function is called first. In this function, the "Move" function will be applied for both vectors and reservoirs. For vector individuals, a search of available reservoir hosts will be launched only if the event "Bite" is selected. Only three fields can be modified during this function: "future X", "future Y", and "next Parasite". After "Step" is done for all host individuals, the "Update" function is applied to each individual. The "x" and "y" fields are filled by "future X" and "future Y" respectively, the "current Parasite" field by the "future Parasite" value with a *1*/*latencyPeriod *probability. The "past Parasite" field is filled by the "current Parasite" value with a *1*/*infectiousPeriod *probability, and birth and death functions are called with *1*/*lifeSpan *probability.

All functions are detailed in the pseudo-code shown in Additional file 1, section 2. All objects re filled from relevant case-studies. Data format is explained in Additional file 1, section 3. The most important point of the data format features remains its capacity to quantify for each habitat a different moving capacity for each vector and reservoir species, leading to the integration of heterogeneous environments. All parameters used in this model are summarized in Table 2.

**Table 2:** Parameters of our model. Each parameter is described in detail in the main text.

**Name**	**Object**	**Comments**
InfectiousPeriod	Parasite	Length (in days) of infectiousness period
LatencyPeriod	Parasite	Length (in days) of latency period
Virulence	Parasite	Extra-mortality (regarding natural lifespan) induced by parasite
OffspringSize	Host	Number of new host produced at each reproduction event
Lifespan	Host	Lifespan of host individual (in days)
BitingRate	Host	Frequency of biting events (in days). Equal to -1 for reservoir individuals.
MovingCapacity	Host	Number of pixels allowd for moving of each side. Leads to a possible moving within a square of *((movingCapacity+1)2)*^*2*^
HabitatTolerance	Host	Number of pixels allowed for moving outside their original habitat.
IndexHabitat	Host	Index of habitat where individual can move without restrictions

## Results and Discussion

### Adaptability examples: Outcomes for modifying system studied

To simplify the identification and modeling of the core mechanisms of our system, we have chosen a simple epidemiological context with only one vector species and one (or two) reservoir species in a landscape with some classes. However the main idea of our model is to be as general as possible. Hence, we developed the possibility to integrate real data coming from real epidemiological situations. In this section, we propose three main (and simple) additions to our basic model. The first one concerns the integration of maps from the Geographical Information System (GIS). This integration allows future studies to integrate real landscapes and to analyze how a pathogen agent can be transmitted within that landscape. However, often, landscapes from GIS require very high resolution that could make impossible the computability of our model. To overcome this problem, we have integrated the implementation of a parallel behavior that transforms our model into a client/server software and allows us to distribute host objects to several computers. Thus, the computability of our model will only depend on the number of available computers. Parallelization makes our model suitable to be used to study disease transmission in really complex environments, and for that reason we can integrate also the last extension which concerns evolutionary dynamics. With the help of these three easy to implement additions, our model becomes a very adaptable tool for the analysis of a wide range of ecological and epidemiological situations.

### Integration of data from a GIS

The way our model has been constructed allows data integration without additional developments. Moreover, as most of the GIS software integrate an output in raster format, we could fill in our habitats, which represent the "virtual world", with the values contained in the GIS output file. This output should indicate values, in terms of habitat classification, for each pixel. Then, as a last step, we would associate each habitat to each vector and reservoir species involved in our landscape.

### Outcomes for intensive computing: Parallelization

When the number of individuals increases too much or when the space to be modeled is too detailed, like in the case of high-resolution maps, computing resources become quickly the limiting factor. To address this issue, we present here a parallel version of our model. This is a first version of parallelization which involves communication between different computers and does not accept a "resume" of the current simulation. Thus different computers can be easily replaced by other chipsets just by substituting communication via "Socket" (a classical method in network programming) by communication via internal memory. The introduction of a "resume" function in a future version of our model should integrate also a better management of unexpected events. Similarly, implementing different moving behaviors requires to adapt the parallelization algorithm.

**Figure 2:**
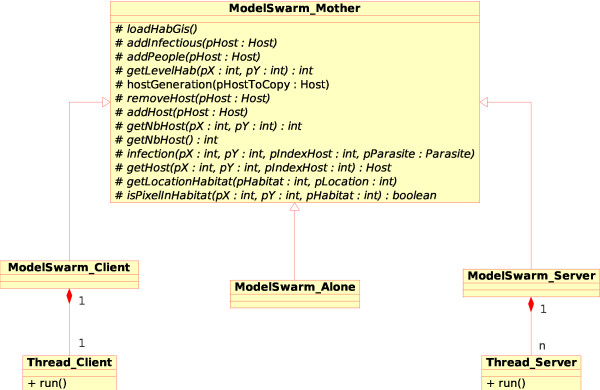
**Distributed behavior of our model**. Each function acting on the virtual world or on scheduling is in the "Model Swarm" class. These are virtual functions and each behavior is implemented in one of the inheriting classes. "Model Swarm Alone" contains the same functions as the "Model Swarm" class and is applied in a stand-alone use. "Model Swarm Client" and "Model Swarm Sever" are called in the case of parallelization. On the server side, a thread is created for each client and communication is done via an established communication protocol (See Additional file 1, section 4). On the client side, each client can focus on a given number of "Host" objects and a thread is created to communicate with the Server component.

This parallelization is quite simple and is based on a classical multi-thread client/server model. As all operations to access the "virtual world" are done by different functions in the "Model Swarm Mother" class, we extended our model to two new classes which inherited from the first one (see Figure 2).

In the "Model Swarm Client" class, each client contains a given number of individuals. These clients have also one thread to receive requests from the server. There are four synchronization messages for clients : (i) "GO STEP", which indicates that the client can launch the "Step" function for each individual ; (ii) "END STEP" which is sent by the client to alert the server when all its individuals have completed the "Step" function ; (iii) "GO UPDATE" which is received by the client to launch the "Update" function for all its individuals and (iv) "END UPDATE" which is sent by the client to indicate to the server that a given time step is completed for that client.

In the "Model Swarm Server" class, upon loading of a GIS file, each new individual is assigned to the less busy client (i.e., the client with the lowest number of individuals). Afterward, a thread is created for communication with each client using the described synchronization messages. When clients need to know some characteristics about one neighboring host or about the habitat value of a given pixel, the server receives a message indicating which value is wanted. A communication protocol has been developed and it is described in Additional file 1, section 4.

This implementation underlines the capacity of an oriented-object program. Indeed, the "Model Swarm Alone" class is used in the case of a non-parallel model. This class inherits from the "Model Swarm Mother" class and it is filled only with "virtual functions" that allow accessing the virtual world or managing task scheduling. These virtual functions will be developed differently for a stand-alone execution, a server execution or a client execution. In this way, we know that the model behavior has not been modified upon parallelization because it is implemented in the Host class and only functions of scheduling and space access can be modified in the parallel version.

### Integration of evolutionary dynamics

So far, evolutionary dynamics of host-parasite interactions in real landscapes have been poorly studied. However, our model could easily cope with this additional complexity. To integrate evolutionary insights, we have to relax the assumption that all disease characteristics are contained in the "Parasite" class. Therefore, we have to model the infection process as a by-product between the host and the parasite. To do that, we have integrated the theoretical framework proposed by Girvan and collaborators [40] to study pathogen evolution. Accordingly, we represented the pathogen and host genomes as binary strings (0 or 1) with a length *n *for the pathogen genome and with a longer string for the host genome. We then computed the Hamming's distance between the two strings to measure the adequation between host and pathogen, and hence the probability of infection. If we then integrate a mutation rate to the pathogen's genome, applied at each new infection, we can also study the evolutionary dynamics of pathogens in space and time. Finally, if we apply also a mutation rate to the host's genome at each new birth, we can analyze the co-evolutionary dynamics as well. This last extension shows once again the adaptability of our model. Just by modifying the "Infection" function and with a conceptual representation of the host and parasite genomes, we can analyze a wide range of evolutionary dynamics in space and time, for and within different vector and reservoir species in a real landscape.

## Conclusion

The model we describe in this paper is one of the simplest for vector-borne diseases. This simplicity, which leads to a model comparable to the classical SEIR stochastic model, allows us to analyze the core mechanisms involved in the spatial features of vector-borne disease dynamics. The analysis of these spatial features (Additional file 1, section 2) show that the spatio-temporal dynamics of vector species are a crucial factor for disease understanding. Moreover, to decrease the intensity of disease transmission, a drastic reduction of vectors' moving capacity has to be applied to produce a significant effect on disease patterns.

The high flexibility of our model allowed us to easily integrate data coming from GIS, thus moving away from theoretical studies to handle real-life situations. However, since real situations need often high-resolution GIS, we have developed a parallel version of our model to cope with all kinds of epidemiological situations. Finally, to strengthen our model's adaptability, we integrated a simple way to study evolutionary and co-evolutionary dynamics.

Of course, our model is not the "magic model" that could be used to model anything, any time and anywhere. Nevertheless, the dramatic increase of vector-borne diseases [41], such West Nile fever, Lyme disease, Chickungunya or others, strongly justifies the generation of this kind of models for a better understanding of complex-system diseases. These theoretical developments have to be continued to fully appreciate the overall impact of spatial features on disease dynamics.

Other spatial modeling methods could be applied as well. The most used model in vector-borne diseases is the reaction-diffusion framework that is really attractive for simple cases. However, when landscape is highly fragmented and the diversity of local habitats could impact on the moving capacity of vector species, tractability of this model could become a problem. Moreover, if the disease studied involves several vector and reservoir species, the number of equations then becomes a critical issue. This limitation also exists for the metapopulation and network models. Hence, the most generic model is probably represented by an individual-based model such as the one we have developed in the present work.

Our main goal is to provide a generic and highly customizable model for vector-borne diseases. Its structure could be applied on a wide range of vector-borne disease, but the different functions and attributes have to be updated to study specific cases. But our model opens new opportunities for the study of infectious diseases. As mathematical epidemiology builds on SEIR framework, we hope that our study has underlined how computational biology could be also well applied to the study of vector-borne diseases. Our model, freely accessible on  under GPL license, is proposed as a first standard version. This model could, and should, be improved to increase the interest in the study of spatio-temporal dynamics of vector-borne diseases in realistic situations.

## Availability and requirements

**Project name**: Tinain

**Project home page**: 

**Operating system(s)**: Platform independent

**Programming language**: Java

**Other requirements**: Swarm platform 

**License**: GNU GPL

## Authors' contributions

BR participated to study conception, carried out the model programming, carried out the analysis of the model and drafted the manuscript. JFG participated to study conception and improved the manuscript. FB participated to study conception, model analysis and improved the manuscript. All authors read and approved the final manuscript.

## Supplementary Material

Additional file 1 Supplementary informations. Biological analyses explained in the main text, all algorithms of the model, procedure for input files loading and communication protocol for distributed behavior. Click here for file
