# Supplementary material for: Multi-agent systems in epidemiology: a first step for computational biology in the study of vector-borne disease transmission
Source: BMC Bioinformatics. 2008 Oct 15;9:435. doi: 10.1186/1471-2105-9-435 (PMC2600827; doi:10.1186/1471-2105-9-435)
Supplement: Additional file 1 — Supplementary informations. Biological analyses explained in the main text, all algorithms of the model, procedure for input files loading and communication protocol for distributed behavior. [file 1471-2105-9-435-S1.pdf]

Multi-agent systems in epidemiology: A first step for computational biology in the study of vector-borne disease transmission

Supplementary Materials

1 Biological analyses

1.1 Dynamical properties of the spatial model: Importance of space

To understand the net impact of the spatial features in a vector-borne disease model, we have analyzed how variations in the moving capacity of vector and reservoir individuals could impact on the intensity of disease persistence. We chose our ecological and epidemiological parameters in order to generate an epidemic as indicated in the legends to figure.

Simulations show that the reservoir moving capacity does not seem to influence the intensity of disease transmission (Figure S1), which is represented here by the maximal number of infectious vectors. Indeed, reservoir individuals are not the organisms that “make contact” between vector and reservoir individuals. If reservoir individuals are within the range of a vector’s moving capacity, the moving capacity of the reservoirs will not change the frequency of contact and hence the probability of infection.

In contrast, vectors’ moving capacity clearly plays a greater role. An increase of vector moving capacity boosts the number of available reservoir individuals that can be reached and bitten. However, a moving capacity’s threshold can be observed (Figure S1). The world used in this example had a 100 pixel size on each side. Although this threshold value is not enough for vectors to reach all reservoir individuals, it seems sufficient, with the support of well-mixed vector individuals, to reach a maximal value in the ratio between vector abundance and reservoir abundance and, thus, to create an epidemic. The existence of a moving capacity’s threshold suggests that vectors’ moving capacity should be reduced below a given threshold to effectively decrease the intensity of disease transmission.

These first results indicate that vectors’ spatio-temporal dynamics may impact more largely on disease transmission than those of reservoir species. However, the majority of the modeling studies on vector-borne disease, like for instance malaria, try to cope with complex modeling of the reservoir host displacements. Our study suggests that it could be better, in the case of vector-borne diseases, to focus on the spatio-temporal dynamics of vector species rather than on the moving characteristics of the reservoir host individuals.

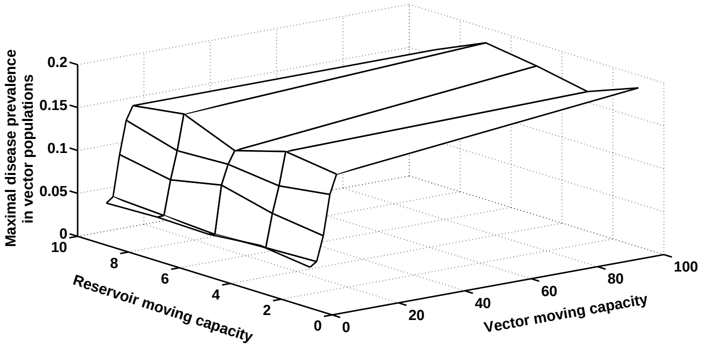

Figure 1: Intensity of disease transmission as measured by the maximal number of infectious vectors, on the basis of different spatial parameters. Reservoirs' moving capacity does not play any role when vectors' moving capacity drives the intensity of disease transmission. When vectors' moving capacity is large, vector individuals can reach a higher number of reservoir individuals. A threshold is shown for a vectors' moving capacity of 10 pixels. With this moving capacity, a given vector individual placed at the center of the lattice can reach a large number of pixels. Hence, many more reservoir individuals will be available for biting. Above a moving capacity of 10 pixels for reservoir individuals, the system is not modified. The lattice size is 100 pixels, abundance of vectors is 1000, abundance of reservoirs is 100, competence values for vector and reservoir species is 80% and 90%, respectively.

1.2 Impact of spatial configuration

After having identified moving capacity's core mechanisms, we could analyze how the spatial distribution of host species influences disease dynamics. Indeed, in natural systems, species are not well mixed as previously assumed, and only some (host) species have contacts with some other (host) species [1].
Here, we looked at which could be the consequences of different spatial configurations in terms of spatial distribution of species in the landscape. Hence, we used a fractal landscape to characterize real landscapes. In landscape ecology, fractal landscape is assumed to be a 'neutral' model [2]. This model does not have a pure formalization of mechanisms. Thus, we decided to use the classical Middle Displacement Point algorithm (MDP [3]) to generate our fractal landscape in order to make it slightly more complex. This fractal landscape yields continuous values and we have to apply an identification cluster every time we want to define a discrete landscape within a given number of classes which will be also analyzed. This operation is classically done in epidemiological studies involving GIS [4]. Finally, to dispatch vector and reservoir species within the fractal landscape, we assumed that vectors are set in odd-numbered habitats and reservoirs in even-numbered habitats.
The surprising result is that neither fractal dimension nor the number of classes seem to have a significant impact on disease dynamics (Figure S2). In fact, despite different spatial configurations, the total number of vector and reservoir individuals is kept constant, and the surface area for each species is similar. In this study, we assume that each individuals could reach any locations within its habitat and its tolerance to go out from its habitat. Hence, the increase of landscape structure complexity decreases area of contacts between individuals located within different habitats, which could, intuitively, decrease disease

transmission.

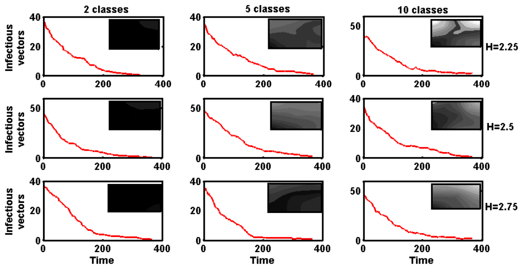

Figure 2: Impact of fractal dimensions and number of possible habitats on disease dynamics. Time series represent the number of infectious vectors across time, and the small inset within each plot illustrates the landscape generated. These two parameters do not impact strongly on disease dynamics: increasing the fractal dimension of landscape and the number of possible habitats will not change the habitat surface area for each vector and reservoir species or the abundance of each species. This phenomenon leads to a constant disease transmission. Parameters used here are identical to those used previously.

1.3 Importance of adding (reservoir) species

The last part of this theoretical study concerns the impact of the addition of a new host reservoir species in space. An interesting effect due to species addition, i.e., the "dilution effect", was found in empirical [5], experimental [6] and theoretical context [7]. The "dilution effect" theory stipulates that disease prevalence in the vector population decreases with the increase of the abundance of low-competent reservoir species. Hence, it might be important to understand how the dilution effect is linked to the spatial dimension.

To this aim, we considered a situation which always leads to disease persistence. We then analyzed how the introduction of one low-competent reservoir species (competence=1%) can buffer disease transmission and lead to lower disease transmission. The size of the newly introduced reservoir species plays a determinant role in disease dilution (Figure S3). Conversely, as it was expected from previous results, an increase of its moving capacity does not. Once again, since vectors "make the contact" in disease transmission, the higher moving capacity of the new reservoir species does not increase the probability for vector individuals to find a poorly competent reservoir individual. On the other hand, a larger population of poorly competent reservoir individuals increases this probability, and hence the "dilution effect" phenomenon.

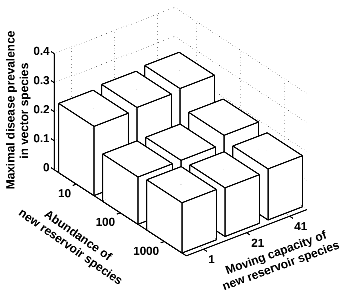

Figure 3: Impact of the introduction of a new reservoir species on the intensity of disease transmission. The population size of the new reservoir species strongly influences disease transmission. This is a direct application of the “dilution effect” as explained previously. On the contrary, the moving capacity of the new species does not influence disease transmission because this parameter does not increase the number of available vectors for each reservoir individual. Parameters used here are identical to those used previously. Competence of the introduced species is 1% and ecological parameters for this species are identical to those of the other reservoir species.

2 Algorithms of function

Host with landscape: Moving function

```
IF BitingRate==-1 OR 1/BitingRate >RAND()
    INTEGER MovingArea=((MovingCapacity+1)*2)^2
    INTEGER IndexNewPosition=CEIL(RAND()*MovingArea)
    NewX=(x-MovingArea+ROUND(IndexNewPosition/MovingArea))
    NewY=(y-MovingArea+ROUND(IndexNewPlace%MovingArea))
    IF NewX AND NewY are not in species Habitat
        NewX and NewY receive closest values in habitat from NewX and NewY
    END IF
    nextX=NewX
    nextY=NewY
END IF
ELSE
    ReservoirList=ModelSwarm.LookingForReservoir(x,y,MovingCapacity,IndexHabitat,HabitatTolerance)
    IF ReservoirList is not empty
        ReservoirChosen=CEIL(RAND()*ReservoirList.size())
        NewX=ReservoirChosen.x
        NewY=ReservoirChosen.y
        Infection(ReservoirChosen.parasite)
        ReservoirChosen.Infection(Parasite)
    END IF
END ELSE
FutureX=NewX
FutureY=NewY
```

Host with parasite: Infection function

```
Parameters : PARASITE pParasite
IF pParasite!=NULL
    IF CurrentParasite==NULL and NextParasite==NULL and PastParasite==NULL
        IF RAND()<Competence
            NextParasite=pParasite
        END IF
    END IF
END IF
```

Main algorithm

```
FOR Time=1 TO Time=TimeMax
    FOR EACH Host Objects
        HostObjet.STEP()
    END FOR EACH
    FOR EACH Host Objects
        HostObjet.UPDATE()
    END FOR EACH
END FOR
```

Function STEP

```
Move()
```

Function UPDATE

```
IF currentParasite==NULL ET nextParasite!=NULL
    IF 1/latencyPeriod <RAND()
        curentParasite=nextParasite
        nextParasite=NULL
    END IF
END IF
IF currentParasite!=NULL ET previousParasite==NULL
    IF 1/infectiousPeriod <RAND()
        previousParasite=currentParasite
        currentParasite=NULL
    END IF
END IF
x=nextX
y=nextY
BIRTH()
DEATH()
```

3 Files input

To fill in all characteristics of host species, parasite species and landscape structure, different input files were needed. The first one was the "Community" file which contains the species characteristics: "Index Species", "Offspring Size", "Lifespan", "Biting Rate" and "Competence" (in order of appearance). Each line represents a new species. The second file was the "Habitat" file which describes the spatial distribution of habitats. Its structure is similar to that of a PPM image file (Portable PixMap file format). The header of the file contains the resolution, in x-axis and y-axis, in the first line, and the number of habitats of the file in the second line. After this header, each line represents a line (in x way) of the picture and pixels on each line are split by tabulation. Finally, the last file was the "Correspondence" file which makes the connection between the "Habitat" and "Community" files. Each line integrates the "Index Species", "Index Habitat", "Moving Capacity" and "Habitat Tolerance" values. Hence, we could take into account the fact that some species have different moving capacities and tolerance values regarding their habitat. This allowed us to characterize the spatial distributions of all species.

4 Communication protocol for distributed behavior

Order	Description
GET PLACE HAB	Get place of host's index in the habitat
GET LEVEL HAB	Get habitat value of input pixel
ADD PEOPLE	Add a new people to the output file for abundance
ADD INFECTIOUS	Add a new infectious to the output file
ADD HOST	Add a new host into the system
GET PIXEL HABITAT	Get habitat value for a given pixel
GET NB	get number of host for a given pixel
GET BITE	Get "BitingRate" value for a given individual
GET HOST	Get "Host" object for a given individual
HOST GENERATION	Create a new "Host" object with input parameters
HOST REMOVE	Remove "Host" object found in input
GET PARASITE	Get "Parasite" object for a given individual, NULL if any
END STEP	Indicating to the "ModelSwarmServer" Object that "ModelSwarmClient" object has finished "Step" function
END UPDATE	Indicating to the "ModelSwarmServer" Object that "ModelSwarmClient" object has finished "Update" function

References

[1] Rozenweig M: *Species diversity in space and time.* Cambrdige University Press 1995.

[2] Turner MG: **Landscape ecology: What is the state of the science?** *Ann Rev Ecol Syst* 2005, **36**:319–344.

[3] Saupe D: *Algorithms for random fractal*, Springer-Verlag 1988 chap. 2, :71–135.

[4] Tran A, Raffy M: **On the dynamics of dengue epidemics from large-scale information.** *Theor Popul Biol* 2006, **69**:3–12.

[5] Ostfeld RS, Keesing F: **Biodiversity and disease risk : The case of lyme disease**. *Conserv Biol* 2000, **14**:722–728.

[6] LoGiudice K, Ostfeld RS, Schmidt KA, Keesing F: **The ecology of infectious disease: effects of host diversity and community composition on Lyme disease risk.** *Proc Natl Acad Sci U S A* 2003, **100**(2):567–71.

[7] Dobson A: **Population dynamics of pathogens with multiple host species.** *Am Nat* 2004, **164 Suppl 5**:S64–S78.
